# Primary headache epidemiology in children and adolescents: a systematic review and meta-analysis

**DOI:** 10.1186/s10194-023-01541-0

**Published:** 2023-02-14

**Authors:** Agnese Onofri, Umberto Pensato, Chiara Rosignoli, William Wells-Gatnik, Emily Stanyer, Raffaele Ornello, Hui Zhou Chen, Federico De Santis, Angelo Torrente, Petr Mikulenka, Gabriele Monte, Karol Marschollek, Marta Waliszewska-Prosół, Wietse Wiels, Deirdre M. Boucherie, Dilara Onan, Fatemeh Farham, Linda Al-Hassany, Simona Sacco, the European Headache Federation School of Advanced Studies (EHF-SAS)

**Affiliations:** 1 Department of Biotechnological and Applied Clinical Sciences (DISCAB), University of L’Aquila, L’Aquila, Italy; 2 Neurology and Stroke Unit, IRCCS Humanitas Research Hospital, Rozzano, Milan Italy; 3 Humanitas University, Pieve Emanuele, Milan, Italy; 4 Department of Clinical and Molecular Medicine, Sapienza University of Rome, Rome, Italy; 5 Wolfson Centre for Age Related Diseases, King’s College London, London, UK; 6 Department of Biomedicine, Neurosciences and Advanced Diagnostics, University of Palermo, Palermo, Italy; 7 Department of Neurology, Third Faculty of Medicine, Charles University and University Hospital Kralovske Vinohrady, Prague, Czech Republic; 8 Department of Neuroscience, Neurology Unit, Bambino Gesù Children’s Hospital, Istituto Di Ricovero E Cura a Carattere Scientifico (IRCCS), Rome, Italy; 9 Department of Neurology, Wroclaw Medical University, Wroclaw, Poland; 10 Faculty of Medicine and Pharmacy, Vrije Universiteit Brussel, Brussels, Belgium; 11 Department of Internal Medicine, Division of Vascular Medicine and Pharmacology, Erasmus MC University Medical Center, Rotterdam, the Netherlands; 12 Faculty of Physical Therapy and Rehabilitation, Hacettepe University, Ankara, Turkey; 13 Department of Headache, Iranian Center of Neurological Researchers, Neuroscience Institute, Tehran University of Medical Sciences, Tehran, Iran

**Keywords:** Child and adolescent headache, Migraine, Tension-type headache, Prevalence, Headache epidemiology, Systematic review, Meta-analysis

## Abstract

**Introduction:**

Headache is the most prevalent neurological manifestation in adults and one of the leading causes of disability worldwide. In children and adolescents, headaches are arguably responsible for a remarkable impact on physical and psychological issues, yet high-quality evidence is scarce.

**Material and methods:**

We searched cross-sectional and cohort studies in Embase, Medline, Web of Science, and Cochrane databases from January 1988 to June 2022 to identify the prevalence of headaches in 8–18 years old individuals. The risk of bias was examined with the Joanna Briggs Institute (JBI) scale. A random-effects model was used to estimate the pooled prevalence of pediatric headache. Subgroup analyses based on headache subtypes were also conducted.

**Results:**

Out of 5,486 papers retrieved electronically, we identified 48 studies that fulfilled our inclusion criteria. The pooled prevalence of primary headaches was 11% for migraine overall [95%CI: 9–14%], 8% for migraine without aura (MwoA) [95%CI: 5–12%], 3% for migraine with aura (MwA) [95%CI:2–4%] and 17% for tension-type headache (TTH) [95% CI: 12–23%]. The pooled prevalence of overall primary headache in children and adolescents was 62% [95% CI: 53–70%], with prevalence in females and males of 38% [95% CI: 16–66%] and 27% [95% CI: 11–53%] respectively. After the removal of studies ranked as low-quality according to the JBI scale, prevalence rates were not substantially different. Epidemiological data on less common primary headaches, such as trigeminal autonomic cephalalgias, were lacking.

**Conclusion:**

We found an overall remarkably high prevalence of primary headaches in children and adolescents, even if flawed by a high degree of heterogeneity. Further up-to-date studies are warranted to complete the picture of pediatric headache-related burden to enhance specific public interventions.

**Supplementary Information:**

The online version contains supplementary material available at 10.1186/s10194-023-01541-0.

## Introduction

Primary headaches, including migraine, are common neurological disorders that represent one of the most prevalent and disabling, although underdiagnosed and undertreated, forms of pain in childhood and adolescence. Comprehensive epidemiological studies on prevalence and incidence of primary headaches in developmental age are lacking and frequently heterogeneous, due to population studies’ characteristics, such as age range, sex, social and economic background, the various methodologies used (e.g., school-based questionnaires, clinician interviews, phone surveys) and the different diagnostic criteria applied, sometimes not specific to developmental age [1]. So, compared to primary headaches in adults, few epidemiological studies are available in children and adolescents, with an estimated prevalence of headache and migraine up to 58% and 7.7% [2] respectively. In the global burden of disease (GBD) of 2016, migraine was ranked first among the most disabling diseases in the 15–49 age range [3]. In children and adolescents, headaches cause a substantial impact on quality of life [4]: limiting social activities, physical activity and school absenteeism, weaker learning outcomes, a higher risk of dropping out of school, and a negative effect on parent's careers [4, 5]. Migraine is also associated with comorbidities such as allergies, sleep disorders, emotional and behavioral problems, depression and anxiety, and academic performances [4, 6].

In this review, we aimed to provide up-to-date information on the prevalence of primary headache disorders in children and adolescents based on a systematic review and meta-analysis of the current literature.

## Methods

### Search strategy

This systematic review was performed according to the Preferred Reporting Items for Systematic Reviews and Meta-Analyses (PRISMA) guidelines [7]. We analyzed all articles published between January 1988 to July 2022 in four databases (Embase, Medline, Web of Science, Cochrane); we also searched for additional references in Google Scholar. The complete search string for each database is available in Supplementary materials.

### Selection criteria

The search aimed to select studies reporting the prevalence of primary headaches in children and adolescents. We selected studies that fulfilled the following inclusion criteria: (i) cross-sectional or cohort study design; (ii) general population or school-based sample; (iii) prevalence of any primary headache in children or adolescents aged 8 to 18 years; (iv) diagnosis of any primary headache according to ICHD diagnostic criteria (any edition). Therefore, we excluded studies including subjects < 8 and/or > 18 years old. Additionally, we excluded studies reporting only epidemiology of secondary headaches or those in which the presence of headaches was addressed in the context of other general medical conditions (e.g. headache in children and adolescents with obesity), or those that dealt with neurodevelopmental disorders such as children with intellectual disability, borderline intellectual disability, psychiatric disorders, attention deficit hyperactivity disorder (ADHD), and tics. We also excluded studies involving highly selected populations (e.g. individuals referred to hospital centers). Review, meta-analysis, case reports, letters, brief or oral communications, and book chapters were excluded unless they provided original data. Studies not published in English were also excluded.

### Article selection methods

Two authors (AO, LAH) performed the search of all electronic databases. The authors AO, UP, CR, WWG, ES, RO, HZC, FDS, AT, PM, GM, KM, MW, WW, DMB, DO, FF, LAH were divided into pairs and independently screened all the titles and abstracts of the studies identified by the initial search for possible inclusion. Following training and exercises to ensure sufficient agreement, reviewers working independently and in duplicate screened titles and abstracts of search records and subsequently the full texts of records deemed eligible at the title and abstract. Conflicts were resolved by discussion and agreement among all the involved authors.

### Data extraction

All the selected studies were entered into an electronic spreadsheet of Microsoft Excel. Included studies were equally and randomly assigned to a pair of authors who extracted the following information: Author, year of publication, country and city, study design (e.g., cross-sectional or cohort), sample size and age range, setting (school-based population/ general-based population), data collection period (expressed in one year or more/months), total sample, age range, females and males (n,%), assessment method (e.g. questionnaire or interview by a clinician), ICHD criteria, total sample headache prevalence and data collection period prevalence (expressed in one year or more/months). When the information was not directly available, it was calculated if possible. All this information was also collected for individual diagnoses of migraine, migraine with aura, migraine without aura, tension-type headaches and unclassified headache, also called undiagnosed or unspecified headache.

### Quality assessment and risk of bias

The quality of included studies was assessed independently by two authors (AO, LAH) using the Joanna Briggs Institute (JBI) critical appraisal tools [8]. A third author (SS) was involved in case of disagreement. The checklist consisted of nine items as follows: 1. Was the sample frame appropriate to address the target population? 2. Were study participants sampled in an appropriate way? 3. Was the sample size adequate? 4. Were the study subjects and the setting described in detail? 5. Was the data analysis conducted with sufficient coverage of the identified sample? 6. Were valid methods used for the identification of the condition? 7. Was the condition measured in a standard, reliable way for all participants? 8. Was there appropriate statistical analysis? 9. Was the response rate adequate, and if not, was the low response rate managed appropriately? Total scores ranged from 0 to 9 and the responses were scored 0 for “No” (red mark) and 1 for “Yes” (green marks). The studies were classified as low-quality, high-risk of bias, if the overall score was ≤ 4.

### Statistical analysis

Continuous variables were presented as means and standard deviations, while categorical variables were presented as counts and percentages. Summary statics were calculated. Statistical analysis of pooled extracted data was performed. Heterogeneity across studies was assessed with Cochran’s *Q* statistics and *I*^2^ statistics. Subgroup analysis was performed based on headache subtypes, namely overall migraine, migraine with aura, migraine without aura, chronic migraine, overall tension-type headache, and unclassified headache. In accordance with the Cochrane Collaboration Guidelines for systematic reviews [9], we assessed the clinical, methodological, and statistical heterogeneity of the studies included. Clinical heterogeneity was assessed by evaluating differences in the populations, exposures, and outcomes. Methodological heterogeneity was assessed by comparing the differences among the adjusted models. Statistical heterogeneity was assessed using the *I*^*2*^ statistic [9]. We performed a sensitivity analysis to quantify the effect of each of the low-quality studies on the overall results. Analyses were carried out with R software [10] using the meta and metapro packages. Whenever the heterogeneity for studies was elevated, random-effect models were applied for meta-analysis. Meta-analysis statistics were performed when at least ten studies were collected, otherwise, summary statistics were implemented. Only data from a subgroup of studies that specified epidemiological results of interest were used among the selected studies included in the meta-analysis. Studies that specifically selected patients with a primary headache subtype through their questionaries, thus excluding patients with headaches not fulfilling distinctive additional criteria, were not included in the meta-analysis of the overall primary headache prevalence (selection bias). These studies were implemented only for the meta-analysis of the primary headache subtypes investigated.

## Results

### Characteristics of included studies

**Fig. 1:**
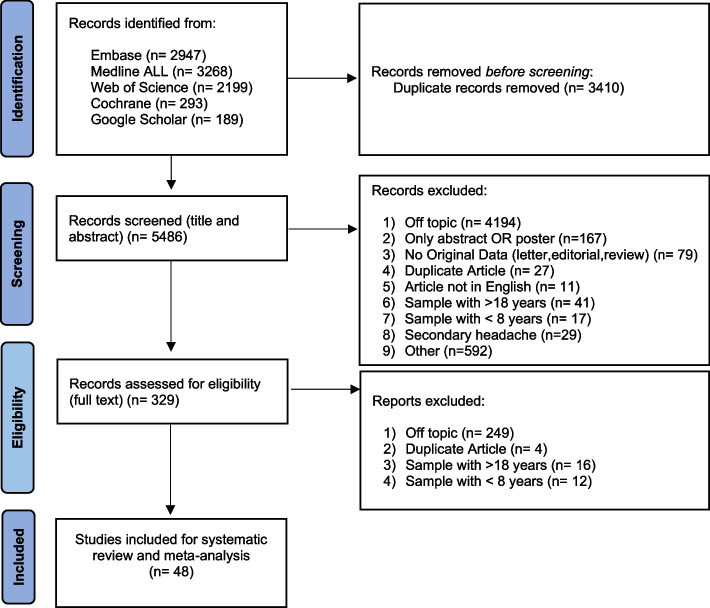
Review process and the reasons for paper exclusion

The systematic search yielded 5,486 papers, of which 329 were found relevant to the topic based on the title and abstract screening. After the full-text assessment, forty-eight manuscripts fulfilled our inclusion criteria. Figure 1 shows the review processes and reasons for paper exclusion.

**Fig. 2:**
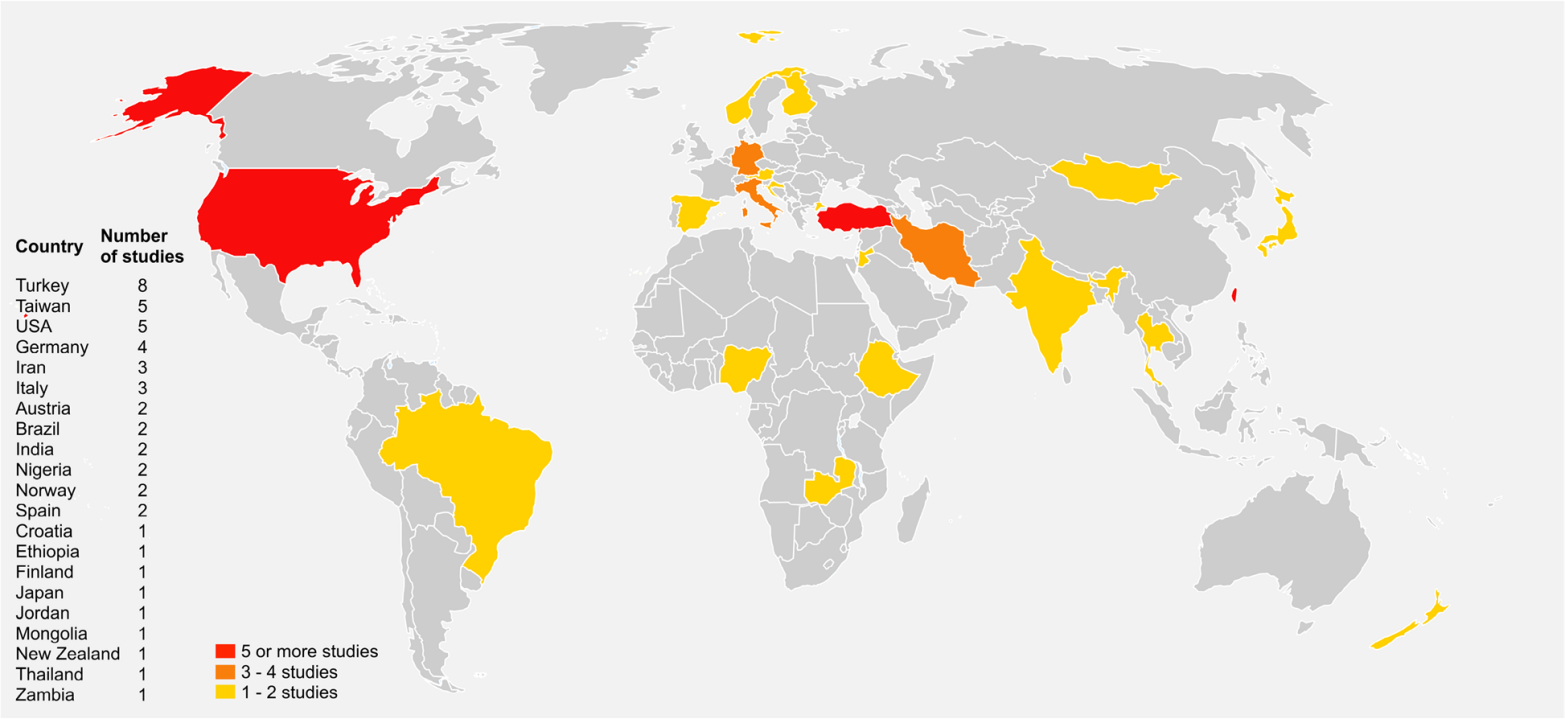
Graphic national representation of the population samples included in each study. *Red—Countries reporting 5 or more prevalence studies; Orange—Countries reporting up to 4 prevalence studies; Yellow—Countries reporting up to 2 prevalence studies*

The worldwide coverage of selected studies is reported in Fig. 2. Most of the studies were conducted in the Middle East (Turkey, Iran), Central Europe (Italy, Germany), and North America (U.S.A.). There was a remarkable underrepresentation of studies from Oceania, Asia, South America, and Africa.

The main characteristics of the studies that reported the prevalence of primary headache are summarized in Table 1. Thirty-six studies that reported the overall prevalence of primary headache in migraine and tension-type headache diagnoses in children and adolescents were included. Nearly 100 percent of the studies had a cross-sectional design (35 studies) and only one was a case–control study. The sample size was quite heterogeneous, ranging from 208 to 9,774 children and adolescents, selected from school-based populations (29 studies), population-based (6 studies) and only one from combination to school-based populations and population-based. Depending on the year of the data collection, the International Classification of Headache Disorders (ICHD), ICHD-1 [11], ICHD-2 [12], or ICHD-3 [13] criteria were adopted to assess each participant for primary headache diagnoses. All studies included in this review used heterogeneous methods for headache assessment. Questionnaire self-report or questionnaire completed from caregivers were the most common methods to identify children and adolescents with headache (22 studies), physician-guided interviews were less common (10 studies), while in the other 4 studies a combination of questionnaire and interview by pediatric headache expert were used.

**Table 1:** Main characteristics of studies reporting overall headache prevalence in children and adolescents. *H: Headache; Q: Questionnaire; I: Interview; ICHD: International Classification of Headache Disorders; M: Migraine; TTH: Tension Type Headache; Y:Yes; N:No*

**Author (ref)**	**Country,** **City**	**Design**	**Target** **Population**	**Data Collection**	**Simple Size**	**Age Range**	**Assessment** **Headache**	**Diagnostic Criteria**	**Prevalence Headache (%)**			**Primary Headache**	
									**Female**	**Male**	**All**	**M**	**TTH**
**Akyol A et al., 2007**[14]	Aydın, Turkey	Cross-sectional	School-based	2004	7721	9–17	Q	ICHD-2	87.1	79.6	83.3	Y	N
**Albashtawy M et al., 2019**[15]	Jordan, Mafraq	Cross-sectional	School-based	2017	754	16–18	Q	ICHD-2	-	-	67.2	Y	Y
**Alp R et al., 2010**[16]	Turkey, Agri	Cross-sectional	School-based	2006	1358	11–18	I	ICHD-2	-	-	34.8	Y	Y
**Ando N et al., 2007**[17]	Japan	Cross-sectional	School-based	2004	6472	12–15	Q	ICHD-2	-	-	59.8	Y	N
**Anttila P et al., 2002**[18]	Finland, Turku	Cross-sectional	School-based	1998	1409	12	Q	ICHD-1	-	-	64.1	Y	Y
**Assadi M et al., 2012**[19]	USA	Cross sectional	School-based	-	309	14–18	Q	ICHD-2	88.0	-	88.0	Y	N
**Barea LM et al., 1996**[20]	Brazil, Porto Alegre	Cross sectional	School-based	1993–1994	538	10–18	Q + I	ICHD-1	94.4	92.3	93.3	Y	Y
**Bektaş O et al., 2015**[21]	Turkey, Ankara	Cross sectional	School-based	2011–2012	5355	9–18	Q	ICHD-2	42.9	35.9	39.4	Y	Y
**Blaschek A et al., 2012**[22]	Germany, Munich	Cross-sectional	School-based	-	1260	15–18	Q	ICHD-2	-	-	83.1	Y	Y
**Bugdayci R et al., 2005**[23]	Turkey, Mersin	Cross-sectional	School-based	-	5562	8–16	Q + I	ICHD-1	52.8	46.2	49.2	N	N
**Cerutti R et al., 2016**[24]	Italy	Cross-sectional	School-based	2013–2014	841	10–16	Q	ICHD-3	32.6	23.7	28.1	Y	Y
**Cvetković VV et al., 2014**[25]	Croatia	Cross-sectional	School-based	2008	2057	14–18	Q	ICHD-2	35.1	25.2	30.1	Y	Y
**Ezeala-Adikaibe B et al., 2017**[26]	Nigeria, Enugu State	Cross-sectional	School-based	2016	218	10–18	Q + I	ICHD-3	53.2	44.9	49.4	N	Y
**Fendrich K et al., 2007**[27]	West Pomerania, Germany	Cross-sectional	School-based	2003–2004	3324	12–15	Q	ICHD-2	78.9	59.5	69.4	Y	Y
**Fuh JL et al., 2010**[28]	Taiwan, Taitung	Cross-sectional	Population based	2005	3955	13–15	Q	ICHD-2	69.9	54.9	62.2	Y	N
**Gupta R et al., 2008**[29]	India	Cross-sectional	School-based	-	2235	12–18	Q	ICHD-2	60.6	55.5	57.5	Y	Y
**Heinrich M et al., 2009**[30]	Germany, Hannover, Saxony	Cross-sectional	Population based	2003–2004	3833	9–14	Q	ICHD-2	68.5	62.0	66.3	Y	Y
**Hommer R et al., 2022**[31]	United States	Cross-sectional	Population based	2001–2004	10,123	13–18	Q + I	ICHD-3	32.2	21.8	26.9	Y	N
**Kaltseis K et al., 2022**[32]	Austria-Italy, North-Tyrol and Bruneck	Cross-sectional	School-based	2015–2018	1923	14–18	I	ICHD-3	56.4	37.6	48.4	Y	Y
**Kawatu N et al., 2022**[33]	Zambia, Lusaka, Copperbelt	Cross-sectional	School-based	2017–2018	1474	12–17	Q	ICHD-3	-	-	87.3	Y	Y
**Krogh AB et al., 2015**[34]	Norway, Sør-Trøndelag	Cross-sectional	School-based	2011–2012	488	12–18	Q + I	ICHD-3	93.8	79.7	88.0	Y	Y
**Lateef T et al., 2019**[35]	USA	Cross-sectional	School-based, Population based	2001–2004	10,123	13–18	Q + I	ICHD-3	16.3	9.4	12.7	N	N
**Lipton RB et al., 2011**[36]	USA	Cross-sectional	Population based	-	24,712	12–17	Q + I	ICHD-2	-	-	39.5	N	N
**Liu HY et al., 2012**[37]	Taiwan, Taitung	Cross-sectional	School-based	2009	663	12–15	Q	ICHD-2	65.8	46.6	56.0	Y	Y
**Lu S et al., 2000**[38]	Taiwan	Cross-sectional	Population based	1998–1999	4064	13–15	Q + I	ICHD-1	87.9	81.3	84.6	Y	N
**Luvsannorov O et al., 2020**[39]	Mongolia	Cross-sectional	School-based	2018	4226	12–17	Q	ICHD-3	-	-	80.7	Y	Y
**Malik HA et al., 2012**[40]	India, Srinagar	Cross-sectional	School-based	-	5000	8–18	Q	ICHD-2	79.3	50.9	66.4	Y	Y
**Ofovwe G et al., 2010**[41]	Nigeria, Benin	Cross-sectional	School-based	2008	1679	11–18	Q	ICHD-2	25.5	14.0	19.5	Y	Y
**Philipp J et al., 2019**[42]	Austria	Cross-sectional	School-based	-	3386	10–18	Q	ICHD-3	82.1	67.7	75.7	Y	Y
**Pothrnann R et al., 1994**[43]	Germany, Wuppertal, Mettmann	Cross-sectional	School-based	1989–1991	4835	8–16	Q	ICHD-1	91.4	87.2	88.1	Y	Y
**Raieli V et al., 1995**[44]	Italy, Palermo	Cross-sectional	School-based	1988–1989	1445	11–14	Q + I	ICHD-1	28.1	19.9	23.9	Y	N
**Turkdogan D et al., 2006**[45]	Istanbul, Maltepe	Cross-sectional	School-based	2003	2504	10–17	I	ICHD-2	-	-	19.3	Y	Y
**Togha M et al., 2022**[46]	Iran	Cross-sectional	School-based	2018–2019	3244	11–17	Q	ICHD-3	72.7	69.0	70.9	Y	Y
**Torres-Ferrus M et al., 2019**[47]	Spain, Catalonia	Cross-sectional	School-based	2015–2016	1619	12–18	Q	ICHD-3	35.1	25.5	30.5	N	N
**Waldie KE et al., 2014**[48]	New Zealand, Auckland	Case–control	Population based	1995–2010	617	11	I	ICHD-3	-	-	42.8	Y	Y
**Zwart JA et al., 2003**[49]	Norway	Cross-sectional	School-based	1995–1997	5847	12–18	Q + I	ICHD-1	84.2	69.4	76.8	Y	Y

### Risk of bias

Table 2 shows all the studies addressed with the JBI critical appraisal tool. No study fulfilled all the quality criteria; thirty-eight ranked above 5 score points (moderate-high quality) and ten ranked below 4 score points (low quality), indicating shortcomings in methodology, in statistical analysis and in sample size.

**Table 2:** Screening parameters, according to the prevalence checklist of related JBI critical appraisal tool and the resulting score for risk of bias of each. *Question codes (Q): Q1—Was the sample frame appropriate to address the target population? Q2—Were study participants sampled in an appropriate way? Q3—Was the sample size adequate? Q4—Were the study subjects and the setting described in detail? Q5—Was the data analyses conducted with sufficient coverage of the identified sample? Q6—Were valid methods used for the identification of the condition? Q7—Was the condition measured in a standard, reliable way for all participants? Q8—Was there appropriate statistical analysis? Q9—Was the response rate adequate, and if it, was the low response rate managed appropriately?*

	**Q1** (sample frame)	**Q2 **(sampling)	**Q3** (sample size)	**Q4** (description of subjects and setting)	**Q5** (coverage)	**Q6 **(identification of condition)	**Q7** (measurements)	**Q8** (statistics)	**Q9** (response rate)	**TOTAL SCORE**
Akyol A, 2007 [14]	√	√	X	X	X	√	X	X	√	4
Albashtawy M, 2019 [15]	√	√	X	√	X	√	√	X	√	6
Alp R, 2010 [16]	√	√	√	√	√	√	√	X	X	7
Ando N, 2007 [17]	√	√	X	X	X	√	√	X	√	5
Anttila P, 2002 [18]	√	√	X	√	X	√	√	X	√	6
Assadi M, 2012 [19]	√	√	X	X	X	√	√	√	X	5
Ayatollahi SMT, 2002 [50]	√	X	√	X	X	√	√	√	X	5
Barea LM, 1996 [20]	√	√	√	√	X	√	√	X	X	6
Bektaş O, 2015 [21]	√	√	√	√	√	√	X	X	√	7
Bigal ME, 2007 [51]	√	√	√	X	X	√	√	X	X	5
Blaschek A, 2012 [22]	√	√	X	X	X	√	√	X	√	5
Bugdayci R, 2005 [23]	√	√	√	√	X	√	√	X	√	7
Buse DC, 2013 [52]	√	√	√	√	X	√	X	X	X	5
Buse DC, 2012	√	√	√	√	X	√	X	√	√	7
Cerutti R, 2016 [24]	√	X	√	X	X	√	√	X	√	5
Cvetković VV, 2014 [25]	√	√	X	√	X	√	√	X	√	6
Ezeala-Adikaibe B, 2017 [26]	√	X	X	√	X	√	X	X	√	4
Fallahzadeh H, 2011 [53]	√	√	√	√	X	√	X	√	√	7
Fendrich K, 2007 [27]	√	X	X	X	√	√	X	√	√	5
Fuh JL, 2010 [28]	√	X	√	X	X	√	X	X	√	4
Gupta R, 2008 [29]	√	X	√	X	X	√	X	X	√	4
Heinrich M, 2009 [30]	√	√	X	X	X	√	√	X	X	4
Hommer R, 2022 [31]	√	√	√	√	X	√	√	X	√	7
Kaltseis K, 2022 [32]	√	X	X	√	X	√	√	X	√	5
Kawatu N, 2022 [33]	√	√	X	X	X	√	X	√	√	5
Krogh AB, 2015 [34]	√	X	X	X	X	√	√	√	X	4
Lateef T, 2019 [35]	√	√	√	√	X	√	√	X	√	7
Lipton RB, 2011 [36]	√	√	√	√	√	X	√	√	X	7
Liu HY, 2012 [37]	√	X	X	X	X	√	√	X	√	4
Lu S, 2000 [38]	√	X	X	√	X	√	√	√	√	6
Luvsanrov O, 2020 [39]	√	√	X	√	√	√	√	X	√	7
Malik HA, 2012 [40]	√	X	√	X	√	√	X	X	√	5
Ofovwe G, 2010 [41]	√	√	X	√	√	√	X	X	X	5
Philipp J, 2019 [42]	√	X	X	√	X	√	X	√	X	4
Pothrnann R, 1994 [43]	√	√	X	√	X	√	√	X	√	6
Raieli V, 1995 [44]	√	√	X	X	X	√	√	X	X	4
Rocha-Filho P, 2014 [54]	√	X	√	X	X	√	√	X	√	5
Saengow V, 2018 [55]	√	√	X	X	X	√	√	X	X	4
Togha M, 2022 [46]	√	√	√	√	X	√	√	√	√	8
Torres-Ferrus M, 2019 [47]	√	√	√	√	X	√	√	X	√	7
Turkdogan D, 2006 [45]	√	√	X	X	X	√	√	X	√	5
Unalp A, 2007 [56]	√	X	X	√	√	√	X	X	√	5
Waldie KE, 2014 [48]	√	X	X	√	X	√	√	X	√	5
Wang SJ, 2006 [57]	√	√	√	X	X	√	√	√	√	7
Wang SJ, 2009 [58]	√	√	X	√	X	√	X	X	√	5
Zewde YZ, 2020 [59]	√	√	X	√	√	X	√	√	√	7
Zwart JA, 2003 [49]	√	√	√	X	√	√	X	X	√	6
Ylmaz M, 2013 [60]	√	X	√	X	X	√	√	X	√	5

### Prevalence of migraine

**Fig. 3:**
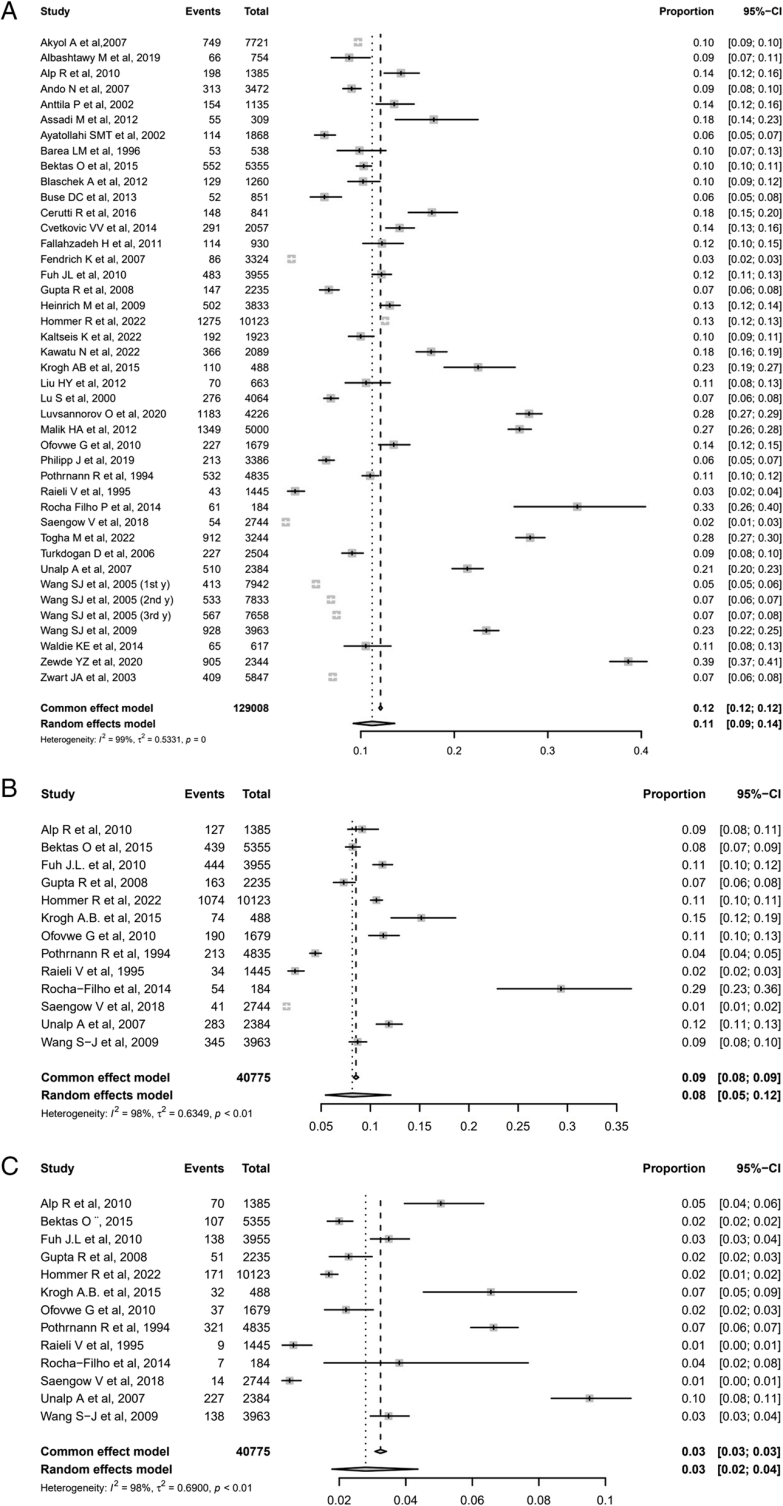
Forest plot of the overall weighted prevalence data of migraine (**A**), migraine without aura (**B**), migraine with aura (**C**). *Total: total population sample; Events: number of primary headache diagnosis; CI: Confidence Interval*

Data were extracted from 40 studies [14–25, 27–34, 37–46, 48–58], including 15,626 children and adolescents with migraine diagnosis. The weighted-pooled prevalence was 11%, the heterogeneity was considerable high (*I*^*2*^ = 98%; 95% CI: 9%-14%). Twenty-seven studies reported the difference prevalence of migraine by sex and the weighted-pooled prevalence of migraine in females was 4% [95% CI: 1%-10%], and 3% [95% CI: 1%-7%] in males. Data for MwoA and MwA were extracted from 13 studies [16, 21, 28, 29, 31, 34, 41, 43, 44, 53–55, 58], including 3,481 and 1,322 subjects with MwoA and MwA, respectively. The weighted-pooled prevalence of MwoA was 8% (*I*^*2*^ = 98%; 95% CI: 5%-12%) and 3% for MwA (*I*^*2*^ = 98%; 95%CI: 2%-4%); again the heterogeneity was ranked as high. See the forest plot of migraine, migraine without aura and migraine with aura meta-analyses in Fig. 3. Data on chronic migraine were reported only by six studies [22, 34, 51, 60–62], with a reported prevalence ranging from 0.2% to 12% (Supplementary Table A).

### Prevalence of tension-type headache (TTH)

**Fig. 4:**
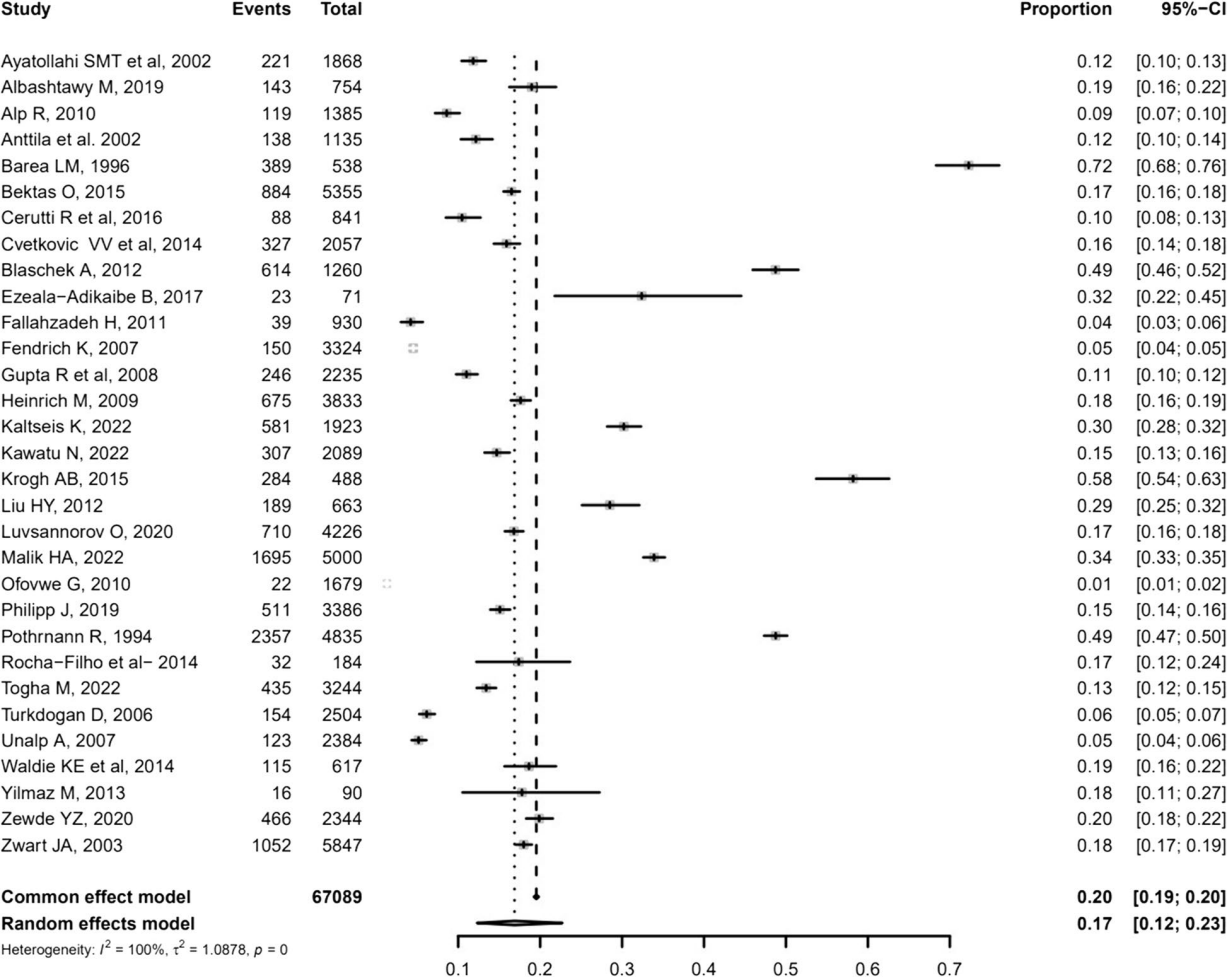
Forest plot of the overall weighted prevalence data of tension type headache. *Total: total population sample; Events: number of primary headache diagnosis; CI: Confidence Interval*

Data were extracted from 31 studies [15, 16, 18, 20–22, 24–27, 29, 30, 32–34, 37, 39–43, 45, 46, 48–50, 53, 54, 56, 59, 60], including 13,105 children and adolescents with TTH diagnosis. The weighted-pooled prevalence was 17%; heterogeneity was considerable high (*I*^*2*^ = 100%; 95% CI: 12%-23%; Fig. 4). Twenty-three studies reported the difference prevalence of TTH by sex; the weighted-pooled prevalence of TTH in females was 11% [95% CI: 5%-22%] and in males was 9% [95% CI: 5%-19%]. Data on episodic and chronic TTH were available only from seven studies [22, 29, 34, 51, 54, 58, 60] and the prevalence was 4–29% and 0.2–12.9%, respectively (Supplementary Table B).

### Overall prevalence of primary headache

**Fig. 5:**
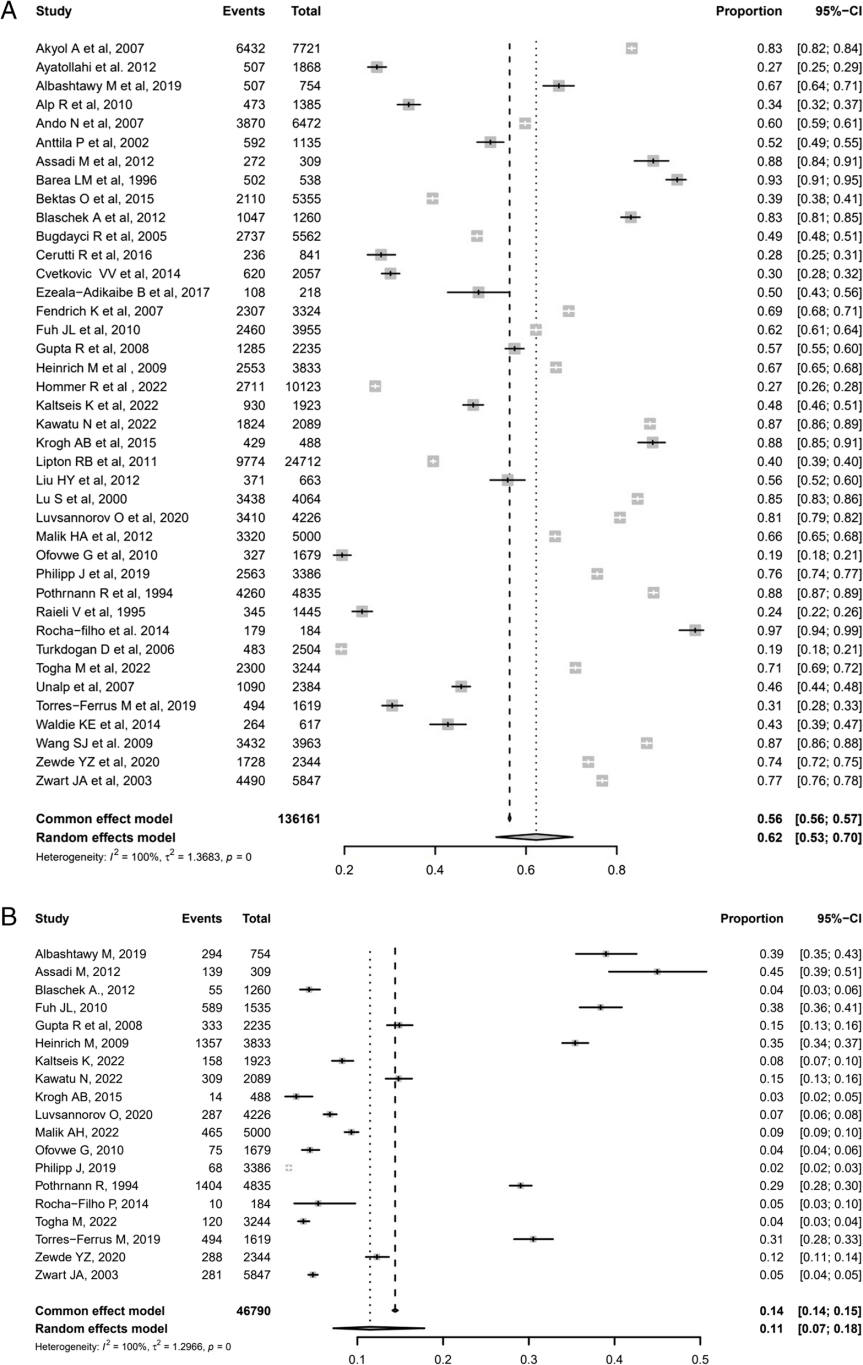
Forest plot of the overall weighted prevalence data of primary headache (**A**) and unclassified headache (**B**). *Total: total population sample; Events: number of primary headache diagnosis; CI: Confidence Interval*

Data were extracted from 40 studies [3, 14–16, 18–23, 25–34, 36–50, 54, 56, 58, 59, 63, 64], including 76,782 children and adolescents with overall primary headache. The overall weighted-pooled prevalence was 62%, heterogeneity was considerable high (*I*^*2*^ = 100%; 95% CI: 53%-70%; Fig. 5A). Twenty-nine studies reported the difference prevalence of overall primary headache by sex; the weighted-pooled prevalence of primary headache in females was 38% [95% CI: 16%-66%] and in males was 27% [95% CI: 11%-53%].

### Prevalence of unclassified headache and other primary headaches

Data were extracted from 19 studies [15, 19, 22, 28–30, 32–34, 39–43, 46, 47, 49, 54, 59], including 6,740 children and adolescents with unclassified headache, also called undiagnosed or unspecified headache. The weighted-pooled prevalence was 11%; heterogeneity was considerable high (*I*^*2*^ = 100%; 95% CI: 7%-18%; Fig. 5B). Data were not found from less common primary headache subtypes, such as trigeminal autonomic cephalalgias (TACs), medication overuse headache (MOH), and new daily persistent headache (NDPH).

### Sensitivity analysis

A sensitivity analysis was performed by excluding studies that were ranked as low-quality (score ≤ 4) at the JBI tool [14, 26, 28, 29, 30, 34, 37, 42, 44, 55]. The results of the sensitivity analysis showed no substantial difference in the overall primary headache prevalence and in the subgroups by diagnosis. In fact, after the removal of the low-quality studies, the prevalence in migraine was 12% [95% CI: 10%-15%], 10% in MwoA [95% CI: 7%-14%] and 4% in MwA [95% CI: 2%-5%], 15% in TTH [95% CI: 10%-22%]. The overall primary headache prevalence in children and adolescents was 62% [95% CI: 50%-71%].

## Discussion

This meta-analysis revealed that the prevalence of migraine in children and adolescents was 11% overall, 8% for MwoA and 3% for MwA. Globally, these data seem to confirm what has already been reported in the literature, where the prevalence of migraine ranges from 7.7% to 9.1% [2, 65] and increases over the course of childhood and adolescence, from 5% among children 5 to 10 years old to approximately 15% among teens [66]. Lack of information in available studies and heterogeneity in stratification by age, limited the possibility to provide pooled data stratified by age and sex. Anyhow, literature data indicated that pediatric headache incidence peaks at 13 years of age [67]. Besides, migraine prevalence rates tend to be similar between boys and girls before 10 years of age, while prevalence increases in females as they approach adolescence [68]. Migraine can cause significant disability in children and adolescents, such as absence from school [69], impaired school performance [4], emotional and psychopathological disorders [5, 70], therefore these data are extremely important and they should be updated constantly. The prevalence of TTH was 17%. The findings are in accordance with the prevalence in the global adult population, in which TTH (38%) is significantly more prevalent than migraine (10%) [64]. Notably, TTH is often diagnosed when the criteria for migraine are not fulfilled, thus, prevalence rates of TTH may be overestimated with a parallel underestimation of the prevalence of migraine. The overall prevalence of primary headache in the pediatric population investigated (8–18 years) was 62%. This is consistent with previous prevalence estimates in children (58.4%) [2] yet higher than previously reported in adults (47%) [64]. Our meta-analysis observed a small increase of prevalence in overall primary headache and in the subtypes headache diagnoses. Several factors could explain this observation, first of all different assessment methodologies for headache diagnosis were used in the studies included. The questionnaires were not always internationally validated and recognized, so the questions did not exactly conform to the ICHD criteria and looked at different time frames (e.g., previous 3–6 or 12 months or lifetime). Also, some studies used self-report questionnaires while other used questionnaires answered by caregivers, especially with younger children. In addition, not all children were referred to a pediatric headache expert, so some diagnoses may have been inaccurate, specially underestimated or overestimated.

### Sex difference in migraine and TTH prevalence

In childhood and early adolescence, boys and girls are equally likely to be affected by migraine, but in late adolescence the prevalence of migraine is higher in girls, with a ratio similar to that seen in adults [2]. Very few studies included in this review and meta-analysis, reported data stratified by sex in the different headache diagnoses analyzed and in overall primary headaches; this notable lack of data could explain the results obtained and thus the high prevalence rates even in the meta-analyses by sex. There was a sex difference in the prevalence of primary headaches (38% for females vs. 27% for males) in accordance with prevalence rates in adults [71]. When analyzing subtypes of headache, this difference was less prominent. For instance, the prevalence of migraine and TTH was only slightly higher in females than in males (4% vs. 3% and 11% vs. 9%, respectively). This aligns with previous studies in which the prevalence is similar between females and males [44, 72], yet is strikingly different from the prevalence in the adult population in which migraine is three times more common in women than men. However, sex differences in migraine prevalence do not typically appear until early adolescence, highlighting the potential role of sex hormones in headache pathophysiology [61, 68]. Notably, reporting of prevalence stratified by sex and age was not consistent in the included studies; hence, we could not stratify our meta-analyses according to definite age groups. Our data are driven by the results obtained in adolescents, in whom the prevalence of migraine is higher in females than in males; in young children before puberty, the relative prevalence of headache in males and females could be very different. Further detailed studies of prevalence according to both age and sex are required.

### Prevalence of chronic headache and other headache

Some primary headache disorders, e.g. migraine and TTH, are well investigated and described in literature, but little is written on many others, especially rare primary headache disorders and the uncommon variants in children and adolescents [73]. Therefore, also in this review, few studies reported prevalence rates for chronic migraine and chronic TTH. There was also a marked absence of data on prevalence of less common headaches such as TACs, MOH and NDPH, which are understudied. Indeed, TACs, including cluster headache and paroxysmal hemicrania, have a low prevalence in the general population and are more commonly observed in the population of adult [63, 64]; MOH and NDPH are also less common in children than adults [18, 33, 65, 66]. The lack of epidemiological studies on the prevalence and incidence of these other primary headaches, demonstrate the need for further studies of less common, though equally disabling, primary headaches in the pediatric population.

### Limits of the studies analyzed

Meta-analyses are valuable tools to collect and analyze the literature on a particular topic using statistical tests, however, any errors or biases finding in the included studies will inevitably affect the analysis [74, 75]. We attempted to address these limitations by testing for heterogeneity and by doing sensitivity analysis to exclude low-quality papers, to ensure the robustness of our results. In this meta-analysis, we included samples from many different countries, although this is limited by including papers only in English. The school-based samples are an effective way to target the representative population, as education is compulsory in most countries, however, a limitation is that these samples cannot account for the children that are not in the education system and are usually affected by poor socioeconomic and health outcomes [76]. Yet, we also included population-based samples, which allowed us to capture a broader pool of participants, including those outside of education. Additionally, the present meta-analysis employs a random-effects model, which is recommended for comparing heterogeneous studies [77], and subgroup analyses to driving factors in prevalence values. This is ideal when dealing with studies with different sample sizes, however, subgroup analyses are limited by the available data, and thus we were unable to compare the effects of factors such as race/ethnicity, socioeconomic factors, geographic background. On the other hand, using ICHD criteria for screening helps to standardize classification across countries, however, it would be interesting to investigate whether the various versions produce different prevalence values. Publication bias also commonly occurs in meta-analyses that collect studies exploring proportions [74], however, in this case, we might consider the possibility that public interest and funding (or lack thereof) may influence research and publishing on primary headaches. These limitations are common across meta-analyses due to clinical and methodological heterogeneity in literature, therefore, taking this into account, the results of this meta-analysis are valuable in informing public bodies on the prevalence and impact of primary headaches on the pediatric population.

### Limits of epidemiology of headache in children

The epidemiology of primary headaches in the pediatric population continues to gain interest, it should be noted that specific findings of high importance remain overlooked. Regarding the limitations of the current literature, there is a great need for high-quality population-based research reporting the epidemiologic variance of primary headaches in age-specific groups [78]. Few articles included in this review reported the implementation of age-specific results in their findings, leaving much to be desired when attempting to postulate the actual burden that primary headaches represent in the younger pediatric population. Besides, most of the included studies only included adolescents and excluded younger children (Table 1); therefore, our results should be taken with caution referring to the youngest age groups, in which headache is often unclassified. Future research should be undertaken to recognize specific pediatric populations that may suffer from increased disease prevalence compared to the overall pediatric population. Isolating age-specific populations at higher risk of primary headache may also provide insight into the potential role of childhood screening and the value of early headache diagnoses to optimize outcomes [79]. Furthermore, the studies were geographically limited, with data lacking from several regions and countries, especially in low-income countries where they are arguably more needed and with possible underrepresentation of non-White ethnic groups. Another area of interest that appears to be overlooked by current literature is the rate at which primary headaches are treated in the pediatric population. It is commonly assumed that primary headaches are under-treated in all populations [80, 81], however, with high-quality data concerning both the rates and efficacy of variable headache treatments, the actual burden of headache within the pediatric population may be accurately assessed [82]. Other important clinical and epidemiological questions that future studies should address are the prevalence stratified by socioeconomic conditions, the headache-related burden in the pediatric population, and the future trajectory of pediatric headache. Indeed, information on the natural history of pediatric headaches would be of utmost importance since early interventions of health policymakers in the pediatric population might significantly contribute to also tackling the gigantic burden of adult chronic headache.

## Conclusion

To our knowledge, this is the first meta-analysis on the overall prevalence of primary headache disorders and sub-types headache disorders in children and adolescents. Collecting data from all prevalence studies of primary headaches in children and adolescents allowed us to suggest a recommendation for the direction of future epidemiological studies. Knowledge of the epidemiology of migraine and other headache disorders has lagged behind developments compared to other areas of neuro-epidemiology. The impact of headache disorders on individuals and society is extensive and provides an important target for public health interventions. Despite the widespread disability produced by migraine, this disorder is still under-diagnosed and under-treated. It is recommended that future research integrating the prevalence, treatment efficacy, and measures concerning the quality of life be undertaken to help elucidate populations at high risk of disease burden. High-quality research of the aforementioned criteria is of great value as it may elucidate novel targets for public health intervention and decrease disease burden in populations that may be overlooked.

## Supplementary Information

**Additional file 1.** Searchstrategy

**Additional file 2.** Supplementary table A and B

## Data Availability

All data generated or analyzed during this review are included in this published article and its supplementary information files.
