# Supplementary material for: Primary headache epidemiology in children and adolescents: a systematic review and meta-analysis
Source: J Headache Pain. 2023 Feb 14;24(1):8. doi: 10.1186/s10194-023-01541-0 (PMC9926688; doi:10.1186/s10194-023-01541-0)
Supplement: Supplementary file 1 — Additional file 1. Searchstrategy [file 10194_2023_1541_MOESM1_ESM.docx]

**Search strategy**

***Embase.com***

(('headache'/de AND ('chronic pain'/de OR 'chronic disease'/de)) OR 'primary headache'/exp OR 'tension headache'/exp OR 'migraine'/exp OR 'new daily persistent headache'/exp OR 'trigeminal autonomic cephalalgia'/exp OR (((primar* OR tension* OR pressure* OR cluster* OR Horton OR new-daily-persist* OR chronic*) NEAR/3 (headache*)) OR ciliar*-neuralgia* OR Horton-syndrome* OR migrain* OR aura OR TTH OR NDPH OR trigeminal-autonomic-cephalalgia* OR hemicrania*):ab,ti,kw) AND ('prevalence'/de OR 'incidence'/de OR 'epidemiology'/de OR epidemiology:lnk OR (prevalen* OR inciden* OR epidemiolog*):ab,ti,kw) AND (child/exp OR adolescent/exp OR adolescence/exp OR 'child behavior'/de OR pediatrics/exp OR childhood/exp OR 'child welfare'/de OR 'child advocacy'/de OR 'child development'/de OR 'child growth'/de OR 'child health'/de OR 'child health care'/exp OR 'child care'/exp OR 'childhood disease'/exp OR (adolescen* OR preadolescen* OR child* OR kid OR kids OR teen* OR boy* OR girl* OR minors OR underag* OR (under NEXT/1 (age* OR aging OR ageing)) OR juvenil* OR youth* OR puber* OR pubescen* OR prepubescen* OR prepubert* OR pediatric* OR paediatric* OR schoolchild* OR preschool* OR highschool*):ab,ti,kw) AND [English]/lim AND [1988-2030]/py NOT ([editorial]/lim OR [letter]/lim OR [conference abstract]/lim)

***Medline***

((Headache/ AND (Chronic Pain/ OR Chronic Disease/)) OR exp Headache Disorders, Primary/ OR (((primar* OR tension* OR pressure* OR cluster* OR Horton OR new-daily-persist* OR chronic*) ADJ3 (headache*)) OR ciliar*-neuralgia* OR Horton-syndrome* OR migrain* OR aura OR TTH OR NDPH OR trigeminal-autonomic-cephalalgia* OR hemicrania*).ab,ti,kf.) AND (Prevalence/ OR Incidence/ OR Epidemiology/ OR epidemiology.fx. OR (prevalen* OR inciden* OR epidemiolog*).ab,ti,kf.) AND (exp Child/ OR exp Adolescent/ OR exp "Child Behavior"/ OR exp "Pediatrics"/ OR "Child Development"/ OR exp "Child Health Services"/ OR exp "Child Care"/ OR "Child Rearing"/ OR exp "Child development Disorders, Pervasive"/ OR (adolescen* OR preadolescen* OR child* OR kid OR kids OR teen* OR boy* OR girl* OR minors OR underag* OR (under ADJ (age* OR aging OR ageing)) OR juvenil* OR youth* OR puber* OR pubescen* OR prepubescen* OR prepubert* OR pediatric* OR paediatric* OR schoolchild* OR preschool* OR highschool*).ab,ti,kf.) AND english.lg. AND 1988:2030.(sa_year) NOT (news OR congres* OR abstract* OR book* OR chapter* OR dissertation abstract* OR editorial* OR letter*).pt.

***Web of Science***

TS=(((((primar* OR tension* OR pressure* OR cluster* OR Horton OR new-daily-persist* OR chronic*) NEAR/2 (headache*)) OR ciliar*-neuralgia* OR Horton-syndrome* OR migrain* OR aura OR TTH OR NDPH OR trigeminal-autonomic-cephalalgia* OR hemicrania*)) AND ((prevalen* OR inciden* OR epidemiolog*)) AND ((adolescen* OR preadolescen* OR child* OR kid OR kids OR teen* OR boy* OR girl* OR minors OR underag* OR (under NEAR/1 (age* OR aging OR ageing)) OR juvenil* OR youth* OR puber* OR pubescen* OR prepubescen* OR prepubert* OR pediatric* OR paediatric* OR schoolchild* OR preschool* OR highschool*))) AND LA=English AND PY=(1988-2030) AND DT=(Article OR Review OR Early Access)

***Cochrane Central Manually***

((((primar* OR tension* OR pressure* OR cluster* OR Horton OR new NEXT daily NEXT persist* OR chronic*) NEAR/3 (headache*)) OR ciliar* NEXT neuralgia* OR Horton NEXT syndrome* OR migrain* OR aura OR TTH OR NDPH OR trigeminal NEXT autonomic NEXT cephalalgia* OR hemicrania*):ab,ti,kw) AND ((prevalen* OR inciden* OR epidemiolog*):ab,ti,kw) AND ((adolescen* OR preadolescen* OR child* OR kid OR kids OR teen* OR boy* OR girl* OR minors OR underag* OR (under NEXT/1 (age* OR aging OR ageing)) OR juvenil* OR youth* OR puber* OR pubescen* OR prepubescen* OR prepubert* OR pediatric* OR paediatric* OR schoolchild* OR preschool* OR highschool*):ab,ti,kw) NOT "conference abstract":pt

***Google Scholar***

"primary|tension|cluster|chronic headache"|"new daily persistent headache"|migraine|"trigeminal autonomic cephalalgia" prevalence|incidence adolescents|children|kids|boys|girls|juvenile|youth|pediatric|paediatric|pediatrics|paediatrics
