# Supplementary material for: Primary headache epidemiology in children and adolescents: a systematic review and meta-analysis
Source: J Headache Pain. 2023 Feb 14;24(1):8. doi: 10.1186/s10194-023-01541-0 (PMC9926688; doi:10.1186/s10194-023-01541-0)
Supplement: Supplementary file 2 — Additional file 2. Supplementary table A and B [file 10194_2023_1541_MOESM2_ESM.docx]

**Suppl.Table A** Prevalence (%) of studies reporting Chronic Migraine by different sex.

|  |  | **CM** |  |  |
| --- | --- | --- | --- | --- |
| **Author (ref)** | ***n*** | **Prevalence (%)** | **F (%)** | **M (%)** |
| Bigal ME, 2004 | 17 | 10 | **-** | **-** |
| Blaschek A, 2012 | 163 | 12,9 | - | - |
| Gupta R, 2008 | 4 | 0,2 | - | - |
| Krogh AB, 2015 | 6 | 1,2 | 1,8 | 0,5 |
| Wang SJ, 2006 | 80 | 1 | - | - |
| Yilmaz M, 2013 | 27 | 5,3 | - | - |

*CM: Chronic Migraine; n=number of children and adolescents with CM; F:Female; M:Male*

**Suppl. Table B.** Prevalence (%) of studies reporting episodic Tension-Type Headache and chronic Tension-Type Headache (cTTH) by different sex.

|  |  | **eTTH** |  |  |
| --- | --- | --- | --- | --- |
| **Author (ref)** | ***n*** | **Prevalence (%)** | **F (%)** | **M (%)** |
| Blaschek A, 2012 | 50 | 4 | **-** | **-** |
| Gupta R, 2008 | 170 | 7,6 | - | - |
| Krogh AB, 2015 | 142 | 29,1 | 26,1 | 33 |
| Rocha-Filho P, 2014 | 29 | 15,8 | - | - |
|  |  | **cTTH** |  |  |
| Bigal ME, 2004 | 17 | 10 | **-** | **-** |
| Blaschek A, 2012 | 163 | 12,9 | - | - |
| Gupta R, 2008 | 4 | 0,2 | - | - |
| Krogh AB, 2015 | 6 | 1,2 | 1,8 | 0,5 |
| Wang SJ, 2006 | 80 | 1 | - | - |
| Yilmaz M, 2013 | 27 | 5,3 | - | - |

*eTTH:* episodic Tension-Type Headache (eTTH); *Chronic Migraine; cTTH:* chronic Tension-Type Headache (cTTH); *n=number of children and adolescents with eTTH and cTTH; F:Female; M:Male*
